# 2443 Attitudes and preferences for return of results from next-generation sequencing

**DOI:** 10.1017/cts.2018.277

**Published:** 2018-11-21

**Authors:** Matthew Neu, Jaimie Richards, Sara J. Knight

**Affiliations:** University of Alabama at Birmingham

## Abstract

OBJECTIVES/SPECIFIC AIMS: Objectives: Decreasing costs and increasing evidence for clinical utility have contributed to whole genome sequencing (WGS) becoming a clinical reality. While previous studies have surveyed the attitudes of patients and community members towards specific gene tests, an emerging literature has begun to describe the preferences of diverse recipients for WGS results. In this study, we sought to identify and synthesize the quantitative evidence on preferences for results from WGS using a systematic review of the literature. METHODS/STUDY POPULATION: We conducted a search of articles on PubMed including subject index terms WGS, whole exome sequencing, genome sequencing, secondary findings, incidental findings, attitudes, preferences, choices, utilities, stated-preferences, discrete choice experiment, and willingness-to-pay. We conducted 11 formal searches to refine the strategy and conducted a final search in December 2017. Duplicates were eliminated and a title and abstract review was conducted to select articles meeting inclusion criteria. RESULTS/ANTICIPATED RESULTS: Our search strategy identified 79 publications meeting initial search criteria with 30 manuscripts meeting inclusion criteria. Of these, most studies were conducted with patient-participants enrolled in existing sequencing studies, while few engaged members of the general public. Of the studies conducted on patients, most were on the medical setting of cancer and related syndromes. The earliest publication date of a manuscript meeting our inclusion criteria was in 2012, yet the majority were published in 2015 or later. DISCUSSION/SIGNIFICANCE OF IMPACT: Between 2012 and 2015, we saw an increasing focus in the medical literature on understanding public and patient preferences for return of results from WGS and WES. Both public and patient populations participating in surveys expressed preferences for receiving results from next-generation sequencing, even if the results are secondary or incidental findings unrelated to the primary indication for sequencing. A primary factor related to patient interest in incidental or secondary findings is the extent to which these results can inform medical intervention. Few studies surveyed representative population-based samples, and this may be an area for future investigation.

